# Zinc Oxide-Based Self-Powered Potentiometric Chemical Sensors for Biomolecules and Metal Ions

**DOI:** 10.3390/s17071645

**Published:** 2017-07-19

**Authors:** Muhammad Israr-Qadir, Sadaf Jamil-Rana, Omer Nur, Magnus Willander

**Affiliations:** 1 Department of Science and Technology, Linköping University, SE-60174 Norrköping, Sweden; miqadir30@gmail.com (M.I.-Q.); omer.nour@liu.se (O.N.); 2 Department of Materials Engineering, School of Chemical and Materials Engineering, National University of Sciences and Technology, Islamabad 44000, Pakistan; 3 Department of Physics, Government College Women University, Sialkot 51310, Pakistan; sjrana54@gmail.com

**Keywords:** zinc oxide nanostructures, self-powered sensor, potentiometric chemical sensors, glucose, cholesterol, metallic ions

## Abstract

Advances in the miniaturization and portability of the chemical sensing devices have always been hindered by the external power supply problem, which has focused new interest in the fabrication of self-powered sensing devices for disease diagnosis and the monitoring of analytes. This review describes the fabrication of ZnO nanomaterial-based sensors synthesized on different conducting substrates for extracellular detection, and the use of a sharp borosilicate glass capillary (diameter, d = 700 nm) to grow ZnO nanostructures for intracellular detection purposes in individual human and frog cells. The electrocatalytic activity and fast electron transfer properties of the ZnO materials provide the necessary energy to operate as well as a quick sensing device output response, where the role of the nanomorphology utilized for the fabrication of the sensor is crucial for the production of the operational energy. Simplicity, design, cost, sensitivity, selectivity and a quick and stable response are the most important features of a reliable sensor for routine applications. The review details the extra- and intra-cellular applications of the biosensors for the detection and monitoring of different metallic ions present in biological matrices, along with the biomolecules glucose and cholesterol.

## 1. Introduction

The enduring advance of sensing technology has progressed alongside the corresponding massive advancements in nanoscience and nanotechnology. The ability of the nanotechnology to operate at the atomic or molecular scale has enabled the miniaturization of smart and portable electronic devices, which was the starting point for acquiring knowledge about the fundamentals of nanoscience leading towards the building of prototype devices and their industrial commercialization [1]. The coming years will be of particular importance as functional devices capable of utilizing the copious amounts of energy available in colloidal solutions are built and these energies are enhanced in the miniaturized nano-environment. The controlled morphologies of nano- and microscale materials are of significant importance in order to overcome the challenges of miniaturization of self-powered energy saving, energy production, and energy harvesting devices by utilizing metal oxide nanomaterials [2,3,4,5,6]. In recent years, the miniaturization of electronic devices has revolutionized the world by facilitating the trend towards portability and increased functionality. The modernization of desktop computers to laptops and tablets, and handheld smartphones to wearable electronics are classic examples which have become everyday necessities for people. 

Metal ions play important roles in living organisms. These ions are extremely useful under particular clinical circumstances and in order to regulate the functions of the immune system, nerves and resting membranes, during transfusion exchange, hemodialysis or organ transplantation, etc. Cholesterol and glucose are also very important biomolecules for various functions in living cells. Variations in the concentration levels of these biomolecules can disturb the production of bile acids, biological signalling, the sex hormones and can cause various deadly diseases, so the use of reliable, robust, and efficient sensing devices to monitor variations in the levels of metal ions as well as these biomolecules is of great importance. 

**Figure 1:**
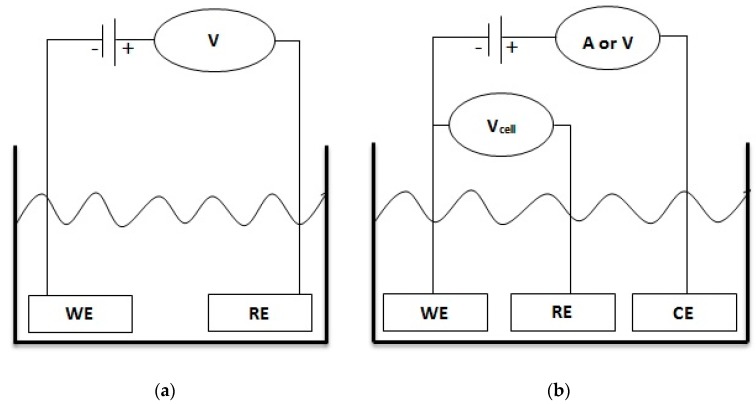
Schematic images of (**a**) a two electrode setup for potentiometric measurements showing its self-powered sensing ability without any external battery/power source, (**b**) a three electrode setup for amperometric/voltammetric measurements with an external power source.

Sensors are typically comprised of four major components; a transducer, a selective layer for the recognition of an analyte, the transducer’s power source, and an electronic signaling system. Self-powered devices are ones which can generate the necessary power to operate through non-resilient variations in a localized environmental chemical energy gradient. Generally self-powered chemical devices don’t produce energy for other purposes but they also do not require any external source for their own operation. Potentiometric chemical sensors are the classic example of self-powered sensors which do not need any external energy source for their operation, because potentiometric sensors are based on two electrode measurement systems, i.e., a working and standard reference electrode, and they can use the chemical energy available in the solution (Figure 1a). The microenvironment in the surroundings of the working electrode created by the accumulation of analytes under the electrostatic mechanism further leads to the production of a potential difference between working and reference electrodes. Other chemical sensors always need an external energy source in the form of current or voltage and are generally comprised of three electrodes, i.e. working, standard reference and counter electrodes, as shown in Figure 1b. 

The starting point for the development of any high quality sensor is strictly related to fabrication of the active material as well as the device design. Inorganic metal oxide nanostructures are a new class of material for the development of functional devices, and they exhibit excellent key features like a stable chemical, thermal and mechanical nature on the nanoscale. Indeed, recently reported metal oxide-based sensors have flourished in the available literature (Table 1) thanks to their crucial features, in particular their enhanced electrochemical signaling [7,8]. Among the various metal oxide nanomaterials, ZnO nanoscale materials are of particular interest due to their excellent properties such as unique nanomorphologies, non-toxicity, catalytic nature, and functional compatibility. Diverse shaped ZnO nanomaterials have been fabricated utilizing different production techniques, e.g., nanofibers through electrospinning, rough nanomorphologies through radiofrequency sputtering, three-dimensional order structures through printing technologies, highly controlled structures through electron beam lithography and molecular beam epitaxy, and various nanostructures through wet chemistry techniques [9,10,11,12,13,14,15,16]. These nanostructures have exceptional capabilities that can be utilized for the fabrication of potentially ideal sensing devices with key features like low detection limits, without the involvement of any filter, ultrafast sensing capability and recovery, strong reproducibility, good sensitivity, high selectivity, and the ability to operate under ambient room temperature conditions. However, in practice most practical devices don’t have the capability to support all the mentioned features above, although a few parameters cannot be ignored to fabricate real time applications. 

Here we will review the fabrication of self-powered chemical sensors based ZnO nanostructures chemically synthesized by aqueous solution-based methods in the Physical Electronics and Nanotechnology Research Group at Linköping University (Sweden), under the leadership of Professor Magnus Willander. Our research group has been working on solution-based syntheses of ZnO nanostructures (nanoparticles, nanorods, nanowires, nanodisks, nanowalls, nanoflowers, etc.) and the use of these structures for the extra- and intracellular sensing of biological species for more than a decade now. 

## 2. Growth of ZnO Nanostructures

As mentioned in the Introduction, the fact that ZnO structures can be grown by a variety of methods is an distinct advantage. As a wurtzite structure, there are three different types of ZnO with a total of 13 growth direction facets together with a pair of polar surfaces which make it a unique structured material. It possesses the best known and richest family of nanostructures. In many applications, the choice of the nanostructure morphology is crucial for the fabrication of the desired nanodevices. In this article, we use different ZnO nanostructures (nanowires, nanowalls and nanoflowers) grown on the variety of substratures (thin silver-wires, boroslicate glass pipettes, aluminum films and zinc foil), where the growth temperature of all the nanostructures is generally >100 °C. Firstly, the substrates were ultrasonically cleaned in ethanol, followed by spin coating a seed solution and annealing at 250 °C for 30 min. For the growth of ZnO nanowires and nanowalls, equimolar 0.1 M concentrations of zinc nitrate hexahydrate (Zn(NO_3_)_2_·6H_2_O, 99.9% purity) and methamine (C_6_H_12_N_4_, 99.9% purity) were used as precursors. The beaker was placed in a preheated oven at 93 °C for 3 h. The nanorods grown on the substrate were taken out of the oven and washed with deionized water and dried at room temperature. ZnO dahlia-flowers on the other hand were grown by dipping zinc foil into formamide solutions with a concentration of 5% for 24 h under nearly ambient room temperature condictions. 

## 3. Chemical Sensors

The first chemical sensor was fabricated around six decades ago, when the existence of chemical reactions within metal oxide surfaces and the surrounding environment were discovered and used for gas sensing purposes. As chemical sensors work as an interface to provide real-time information about various changes occurring in their surroundings, and such information can be related to the detection of different molecules by spreading out these sensors, this can help us monitor variations in hazards due to chemicals and radiation, environmental toxicity, humidity variations, health conditions, food quality control system, and outdoor monitoring. Additionally, workplace monitoring for the safety of workers has become a very prominent issue in industry. In recent years, not only has awareness about physical, mental and psychological fitness increased considerably, but also it means a lot for the common people who aim to enjoy a healthy everyday life in good spirits. In order to fulfill the requirements for viable practical applications, the sensing devices used need to be efficient, reliable, selective and stable. The effectiveness of early diagnostics and cures for diseases is becoming more and more important in various aspects in our routine life. Self-powered potentiometric sensing measurements are being performed using two-electrode measuring systems, where one of them is a working electrode based on a ZnO nanostructure encapsulated with a selective membrane, and second one is a standard reference electrode (Ag/AgCl). The potential difference among these electrodes can be utilized to measure the concentration levels of biological species without applying any external potential.

## 4. Enzyme Immobilized ZnO-Based Biosensors

The combination of ZnO nanostructures with enzyme redox reactions is a novel strategy for the detection of various important biomolecules in living bodies. However, it is more important to control the elevated or diminished levels of such biomolecules in the blood stream, as these variations can be responsible of the prevalence of serious diseases in living beings [17,18,19]. Such biosensors have repeatedly shown excellent prospects in the recognition of the biological events as well as their transduction into electrical signals. The large difference in electrostatic surface potential between ZnO and enzymatic materials is an important factor for formation of the electrochemical interface. The strong electrochemical interfaces between nanostructures and enzymes effectively improve the signal transfer rate, detection limit, sensitivity and selectivity for biomolecules.

## 5. Cholesterol Sensors

Cholesterol is a very important biomolecule that is crucial for various functions in living cells. A controlled cholesterol concentration level in the bloodstream is very necessary because this balanced level of cholesterol can help ensure the correct functioning of the organism, such as the production of bile acids and sex hormones, signalling processes, etc. [20,21]. Determination of cholesterol levels in the bloodstream is of vital importance, and it can be accomplished through the chemical reaction between the enzyme cholesterol oxidase and the oxygen present in buffer solutions. 

According to the available literature, the bifunctional nature of the enzyme cholesterol oxidase is responsible for the initiation of the reaction its function as a catalyst in order to produce a Δ^5^-3-ketosteroid along with hydrogen peroxide through an oxidation process (Equation (1)). Subsequently, the Δ^5^-3-ketosteroid undergoes an isomerization process immediately after the completion of first step and a transition of the intramolecular proton between the 4β and 6β arrangement. A stable Δ^4^-3-ketosteroid forms due to the special ability of cholesterol oxidase enzyme where it can produce a specific chemical modification of the steroid in the presence of the 3β-hydroxyl group (Equation (2)) [22]. The complete reaction process is shown in the following equations:

Cholesterol + O_2_ → Δ^5^-3-ketosteroid + H_2_O_2_ (1)

Δ^5^-3-ketosteroid → Δ^4^-3-ketosteroid (2)

**Figure 2:**
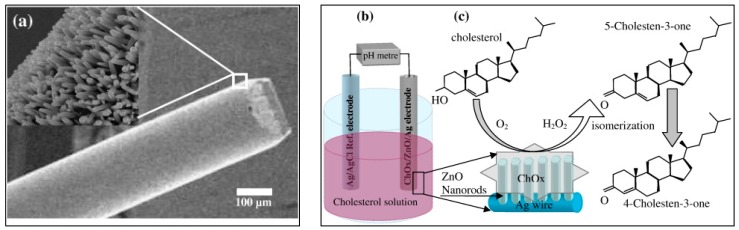
(**a**) SEM image of ZnO nanowires chemically grown on a silver wire. (**b,c**) Experimental setup and reaction mechanism of the cholesterol detection process [23].

Israr et al. reported the fabrication cholesterol biosensor using ZnO nanowires grown on a thin silver microwire where the grown nanowires were highly oriented with respect to the wire surface [23]. A scanning electron microscope (SEM) image is shown in Figure 2a. A schematic of the electrochemical potential difference measurement between the cholesterol biosensor vs a standard reference electrode and the suggested mechanism along with the resulting changes in the cholesterol molecule can be seen in Figure 2b,c. This is the same chemical reaction process mentioned in Equations (1) and (2) and considered responsible for the generation of a potential in the surroundings of the fabricated biosensor. The high surface area to volume ratio of nanowires was considered as one of the most important parameters to achieve an excellent sensitivity curve of ~35.2 mV per decade, proving the improved values of the potential in the surroundings of the cholesterol biosensor with increasing concentrations of cholesterol buffer solutions. The electrochemical response of the measured results revealed the excellent sensing capability over a broad logarithmic scale range from 1 × 10^−6^ M to 1 × 10^−2^ M cholesterol concentrations (results not shown here).

**Figure 3:**
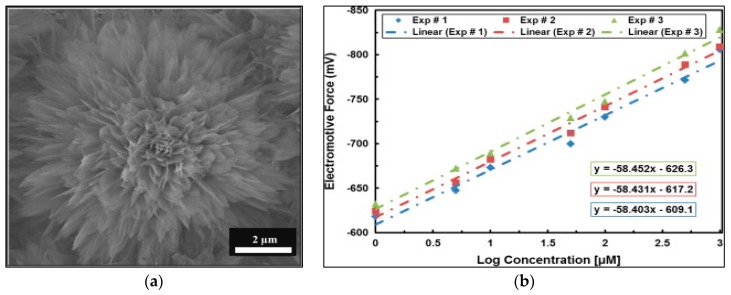
(**a**) SEM image of three-dimensional ZnO dahlia-flowers. (**b**) Repeated calibration curves for the cholesterol biosensor against a standard Ag/AgCl reference electrode [24].

Another report [24] disclosed chemically grown ZnO nanoflowers comprised of very thin two-dimensional petals on a zinc foil substrate, shown in Figure 3a. The electrochemical response of the biosensor reveals a good sensing nature with an electrochemical response of ~58 mV per decade along with excellently repeatability and reproducibility capabilities in repeated experiments where it was evaluated for a slightly different concentration range of 1 × 10^−6^ M to 1 × 10^−3^ M buffer solution. The fabricated biosensor was washed in pH 7.4 phosphate buffer saline solution in order to ensure the absence of any possible residue on its surface before the next measurement. Additionally, the biosensor was also checked for its reusability and it was found that the behavior of the electrochemical response curve was same in each measurement, with negligible changes, as shown in Figure 3b.

**Figure 4:**
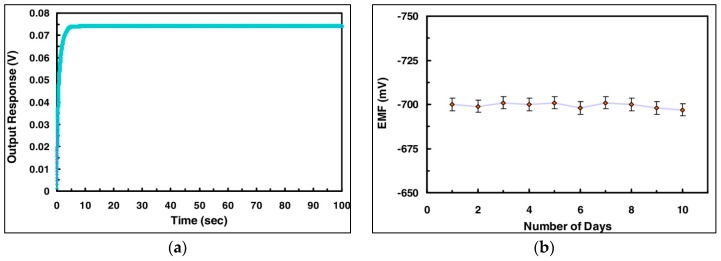
(**a**) Output response for the cholesterol biosensor against the reaction time. (**b**) Storage capability of the biosensor repeatedly tested for 10 consecutive days [25].

Generally, the output response with a stable signal is very quick for ZnO encapsulated cholesterol oxidase sensors and lies in the range of 2–20 s for different nanostructures against some specific and suitable concentration of the buffer solution. Here, we can see the output response as well as the stable nature of the enzyme-based sensors, and the storage abilities along with the reusability were checked. Figure 4a shows the results of the biosensor related to the electrochemical response and its stability against a 100 μM concentration of cholesterol buffer solution and its stable response of ~5 s [25]. The highly increased surface of the nanostructures as compared to their counterpart bulk material is considered responsible for the quick and stable response of the biosensor in the buffer solution because the extended surface of the biosensor allows the signal communication between the active nanomaterial matrix and the assayed cholesterol. The biosensor was also tested by performing experiments for 10 consecutive days and it was found that the biosensor’s results were almost unchanged as shown in Figure 4b [25]. The slight variations in the electrochemical response could be related to the experimental setup, and the biosensor was stored in a suitable environment prior and after the experiments. Table 2 shows a comparison between externally powered chemical sensors (EPCS) or self-powered chemical sensors (SPCS) fabricated by different materials.

## 6. Glucose Sensors

Glucose is an essential carbohydrate and a crucial energy source in the biological systems of humans and other organisms. It is fundamental ingredient of the fat produced in the metabolic system. As it is a very necessary component of the human body, researchers have therefore focused on the fabrication of glucose biosensors in order to monitor glucose levels in the blood. One of the best possible techniques to determine the average value of glucose for a group of cells is nuclear magnetic resonance. This technique is a nondestructive and an indirect way to gather information about the concentration of glucose in the blood. For direct measurement of glucose concentrations, biosensors based on ZnO nanomaterials are being considered as good alternative to the present methods [35,36]. Our research group has published several articles related to the fabrication and application of ZnO nanostructure-based glucose biosensors, not only for extracellular but also for intracellular measurements to achieve precise, accurate and highly resolved results for real-time applications. Such reports on intracellular measurements are not very common in the literature [37]. The reactions for the detection of the glucose molecule with ZnO/glucose oxidase enzyme-based sensors are given in the following equations:

H_2_O_2_ + O_2_ + β-d-glucose → δ-d-gluconolactone + H_2_O_2_ (3)

δ-d-gluconolactone → d-gluconate^−^ + 2H^+^ (4)

The reaction is highly specific due to choice of the analyte and the glucose oxidase enzyme which has an excellent functionality with products such as δ-gluconolactone along with hydrogen peroxide. The combination of these two products and the consumption of the oxygen in the solution works to produce an electronic signal for the detection of glucose molecules. The chemical reaction step occurs when the gluconolactone reacts with water at neutral pH and converts into the ionic form, gluconate ion, and protons, as shown in the equation above. 

**Figure 5:**
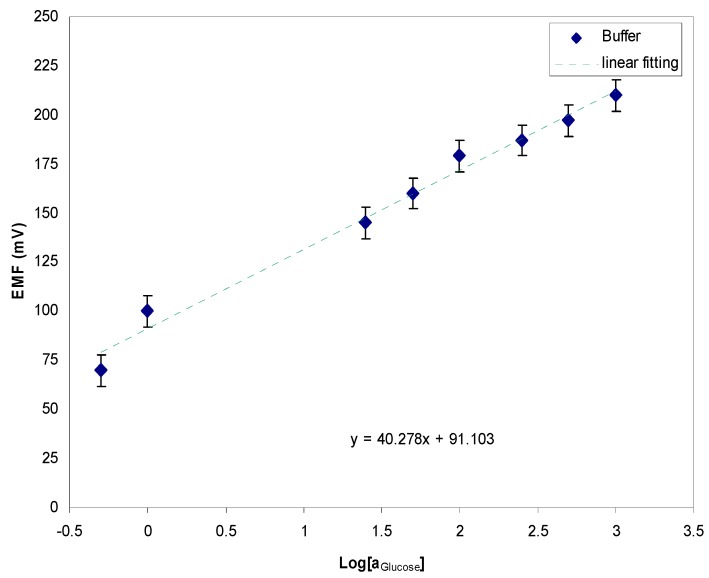
The electrochemical response of microscale biosensor against the logarithmic range of glucose electrolyte [38].

Asif et al. reported the fabrication of a ZnO-based microscale biosensor and detection of glucose molecules over the concentration range from 500 nM to 1 mM of buffer solution, and tests of the electrochemical response against a standard reference electrode. The electrochemical curve displayed a linear sensitivity response of 40.2 mV per decade, as shown in Figure 5 [38]. The results obtained for human adipocytes (50 ± 15 µM) were comparable to the results obtained for rat muscle tissues for an extracellular glucose concentration of 10 nM [39]. A stable potential response was obtained for the intracellular measurements after the injection of 10 nM insulin as an additional amount of extracellular solution, where the result measured intracellularly in frog oocytes was 125 ± 23 µM. Increased values of the glucose concentrations (50 ± 15 to 125 ± 15 µM), and (125 ± 23 µM to 250 ± 19 µM) have been noticed for human adipocytes and frog oocytes, respectively, a few minutes after the injection of insulin. 

**Figure 6:**
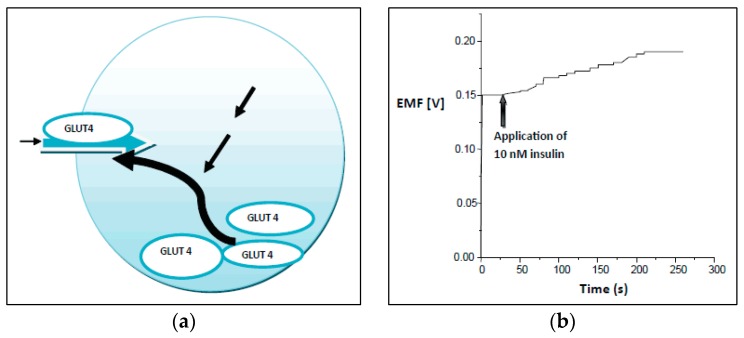
(**a**) Mechanism of the insulin-induced activation of the glucose uptake. (**b**) Impact of insulin induction in the extracellular solution on the output response curve during intracellular glucose sensing measurements [38].

Measurements obtained utilizing functional ZnO nanorod-based biosensors revealed highly consistent results for the glucose values present in human adipocytes and frog oocytes, as shown in Figure 6a,b. Another report [35] focused on glucose detection with ZnO nanoflakes as an alternative to ZnO nanorod structures; the effort was made to synthesize the ZnO nanoflakes directly on a sharp borosilicate glass tip for gentle penetration into the cell membrane. Improved results with better selectivity and sensitivity, with a lower detection limit and higher efficiency were claimed due to the higher surface area to volume ratio.

Another feature of this approach can be the potential existence of microcavities which can grab higher quantities of the glucose oxidase membrane which is a key parameter, not only to detect the biomolecule but also for the selectivity, sensitivity, and efficiency of the measurements. 

## 7. Ionophore Immobilized ZnO Based Sensors

Metal ions are extremely important in terms of their physical, chemical and biological functions in living systems [40,41]. In living species these cations can influence nervous impulses, vesicle exocytosis apoptosis, the immune system, neuronal and enzyme activities, the modulation of ionic transport in nerves and salt homeostasis and several other functions [42,43,44,45,46,47].

## 8. Zinc Ion Sensors

**Figure 7:**
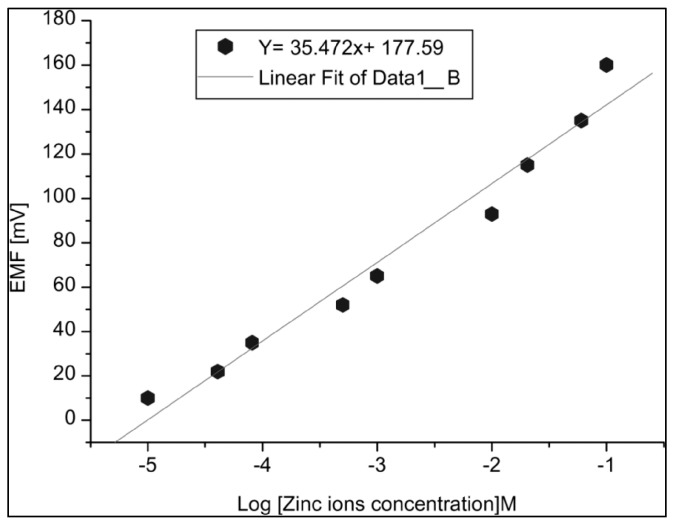
Calibration curve for zinc ion detection using ionophore-coated ZnO nanorods [48].

Zinc is an important cation in biological systems that participates in several functions, so it is necessary to monitor the concentration of zinc ions in the blood. This check on the varied concentrations of Zn^2+^ can have positive impact on gene expression, immune system function and the efficiency of enzymes. Zafar et al. presented results about the suitability of a ZnO nanorod-based sensor for the detection of Zn^2+^, where the focus was to fabricate a Zn^2+^-selective biosensing probe for the electrochemical response of the fabricated sensor [48]. The ability of the ZnO nanorod-based biosensing probe encapsulated by ionophore membranes was verified through the monitoring of in vivo measurements of biological species [42]. The electrochemical response curve for varying concentrations of Zn^2+^ from 1 µM to 100 mM in phosphate buffer solutions was drawn to test the sensitivity of the biosensor, and the measured results revealed the good linear response of ~35 mV per decade under ambient temperature conditions (23  ±  2)  °C, as shown in Figure 7. The following equation shows the representation of the electrochemical potential cell reaction which was suggested in this work:

[Ag|ZnO|buffer||Cl^−^|AgCl|Ag]

Various parameters of the fabricated sensor were evaluated like its detection limit, response time, reproducibility, repeatability, etc. The presented study considered the reproducibility of the device as an important parameter, so a set of five independent sensors was fabricated keeping fixed all other acquisition parameters, including the ionophore membrane. The ionophore membrane contains O, S, N, etc. which are not only considered as a key elements responsible for generating an electrostatic interaction between Zn^2+^ and the active ZnO matrix but also for the stability of the device for a longer period of time.

**Figure 8:**
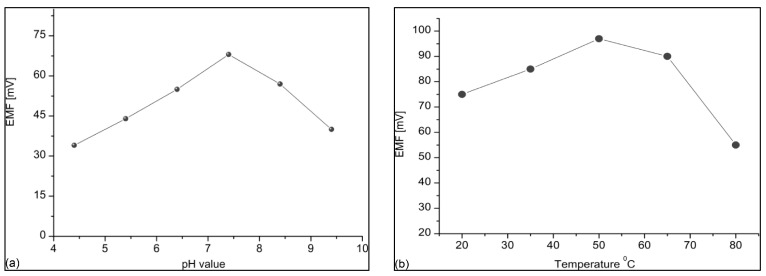
(**a**,**b**) Impact of different pH and temperature values on the response curves of zinc ion sensor [48].

The impact of the varied values of pH and temperature of the phosphate buffer solution was also investigated with the aim of illustrating the ability of the sensor under any particular environmental condition. The authors suggested that the surface of the ionophore membrane is not highly stable when exposed to a varied ionic distribution like that found in an acidic or basic environment and the electrochemical response starts to decline as the pH value moves away from neutrality. The highest value of the electrochemical potential response was found in the neutral pH range of 7–8; such results have also been reported in the literature by other authors [49]. Figure 8a shows the results drawn from the ZnNO_3_ solution prepared in phosphate buffer solution, which was used to test analytical solutions for the pH range from 4 to 10 and best results are shown at the pH value of 7.4. The impact of high temperature was observed for the range between 20 and 80 °C, the trend of the measured curve is in the upward direction until the temperature value of the solution reaches 45 °C; however, it declines sharply as it crosses the 50 °C limit, as shown in Figure 8b. 

The degradation in the electrochemical response is related to the biodegradability at higher temperature and weakening of the binding forces of the membrane. Additionally, the increase in the kinetic energy of the other ions present in the solution can prevent the Zn^2+^ from accessing the surface of the biosensor. 

## 9. Magnesium Ion Sensors

**Figure 9:**
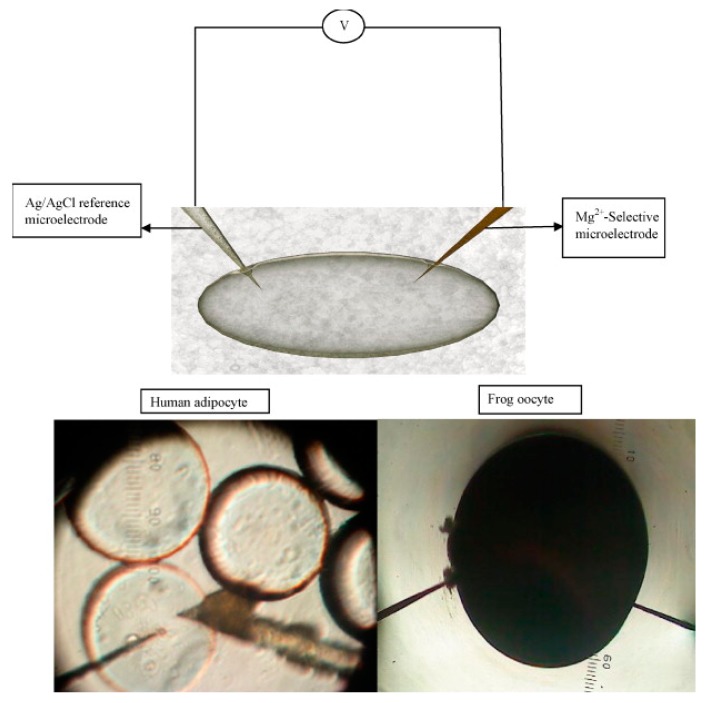
Schematic image of the two electrodes measurement setup. Microscopic images of human cells (adipocytes) and a single frog cell (oocyte) [52].

Magnesium is the cation with the highest percentage (98% of its total amount) available in the intracellular fluid and it works to regulate the nerves, resting membranes, and the physiological functions of the muscles [50,51]. However, the detection of magnesium is very hard, so the fabrication of a highly selective, sensitive and efficient sensor for this species is an attractive area of research. Mg^2+^ concentrations in frog oocytes (0.4 to 0.5 mM) and human adipocytes (0.8 to 0.9 mM) were investigated through the measurement of electrochemical potential utilizing a ZnO nanorod-based biosensor. A two electrode measurement setup was utilized for investigation at ambient room temperature and both electrodes were inserted into the cells to measure an electrochemical potential, as shown in Figure 9 [52,53,54].

**Figure 10:**
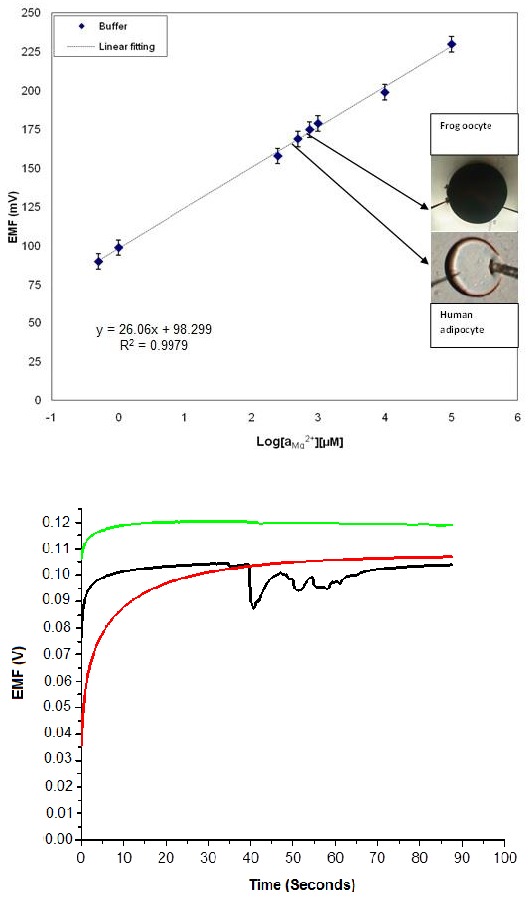
(**Top**) Calibration response curve of the Mg^2+^ sensor vs. reference electrode. The inset shows images of frog and human cells. (**Bottom**) The output response curves without (red) and with (black and green) interfering ions in the analyte solution [52].

A highly selective and stable output response has been achieved in buffer solution with (black/green) and without (red) the presence of interfering ions, as shown in Figure 10. The stability and selectivity of the sensor was investigated in the presence of interfering ions for 1 µM concentration of buffer solution (Figure 10). The human adipocyte cells utilized to probe the sensitivity and selectivity investigations of various concentrations of Mg^2+^ were isolated from collagenase digestion of subcutaneous adipose tissue pieces, and were collected through elective surgery at Linköping University Hospital, Sweden [55]. The ovarian lobes were collected from abdominal incision of female *Xenopuslaevis* after anesthetization. Similarly, the ZnO nanorod-based sensor was optimized for selective e.g., Na^+^ and K^+^ detection for its use in intracellular environments. The EMF response for Na^+^ in buffer solution against varied concentrations ranging from 500 nM to 100 mM is depicted in a sensitivity curve with 72 mV per decade at 23 °C (Figure 10). Na^+^ is another important cation which helps to regulate mineral levels as well as their reabsorption in the kidneys; and it has several other as yet unelucidated functions as well [56,57]. 

## 10. Calcium Sensors

**Figure 11:**
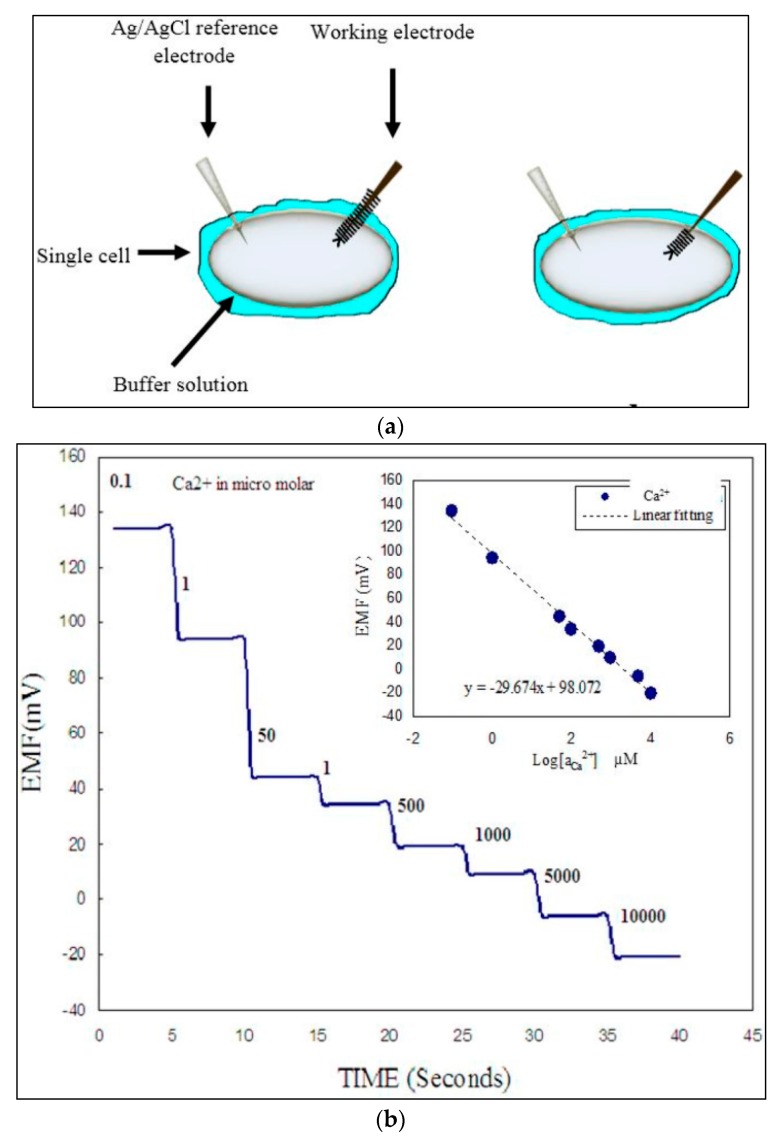
(**a**) Schematic image of a micro-electrode partially and completely inserted in a cell. (**b**) The electrochemical response of the partially inserted micro-electrode with varied concentration of buffer solution in the surroundings. Inset shows the response curve of a biosensor completely inserted in the cell [42].

The presence of calcium ion in the body is an essential compartment, and in vivo monitoring of this species to prevent any sudden fluctuation in Ca^2+^ concentration is also important under many special clinical circumstances, for example during transfusion exchange, hemodialysis or organ transplantation [58]. Sensing measurements were carried out on a single cell by varying the Ca^2+^ concentration range in phosphate buffer solution from 100 nM to 10 mM in the surroundings of the cell. As shown in Figure 11a, two different mechanisms were chosen to measure the results; first, the functionalized part of the biosensor was partially inserted into the cell while the other half was in contact with the phosphate buffer solution surrounding the cell. The results showed a stepwise potential response in the electrochemical measurements as the concentration of buffer solution changed in the surroundings of cell, as shown in Figure 11b. However, in the second case a very stable potential response was measured which was independent from any changes or variation in the concentration of the buffer solution, giving the linear response curve shown in the inset. 

This stable signal response is highly in line with the measured value of the Ca^2+^ concentrations for single human and single frog cells reported in two previous studies which are (123 ± 23 nM) and (250 ± 50 nM), respectively [59,60]. It is also important to know the precise concentration of the buffer solution in surroundings of the cell to get reliable measurement results, so the measurement of the electrochemical response was carried out for the concentration range from 100 nM to 10 mM, as shown in inset of Figure 11b [42].

## 11. Concluding Remarks

This review article deals with self-powered chemical sensors fabricated for the detection of biomolecules and metal ions present in extra- and intra-cellular solutions. These devices take their operational energy from the electrocatalytic activities of the metal oxide nanostructures and the application of such nanomaterial-based devices is surely expanding continuously. The amount of available energy has a strong dependence on the morphology of the materials used for the device fabrication and the transducer used for the selectivity of the analyte. In this review, the synthesis of diverse ZnO nanomorphologies on conducting substrates for the extracellular detection of various biological species has been described. Unique biosensing devices on thin sharp borosilicate glass capillaries have also been fabricated for intracellular detection purposes. These devices have been tested on individual human and frog cells, allowing very specific and highly resolved information about biomolecules to be obtained. However, other parameters like simple fabrication processes and the design, reliability, portability, and affordability of the biosensors are also highly desirable features for consumers. These devices have shown very quick (1–2 s) linear responses over a broad analyte concentration range along with robust, stable response, highly selective and sensitive natures. These devices are highly recommendable for the diagnostic and monitoring purposes for a variety of diseases, and it seems only a matter of time before these devices will become available for real time application in routine life. 

## Figures and Tables

**Table 1:** Metal oxides with their isoelectric points (ISE), important features, and target molecules for biosensing applications (ChOx, Cholesterol; GOx, Glucose; IgG, Immunoglobulin; Urs, Urea; MIOs, Metallic ions).

Materials	ISE Point	Important Features	Target Molecules
TiO_2_	3.9–8.2	Biocompatible, photocatalysis	ChOx, Urs, DNA, IgG
V_2_O_5_	3.0	Catalysis	DNA
SnO_2_	4.5–5.0	Low working potential, electron transfer	ChOx, GOx, DNA, IgG
Fe_3_O_4_	8.4–8.5	Superparamagnetism with O_2_ atoms of proteins	ChOx, GOx, Urs, DNA
SiO_2_	1.7–3.0	Biocompatible, functional	ChOx, GOx, DNA, IgG
CuO	9.5	Multi-electron oxidation	IgG
CeO_2_	6.7–9.5	Biocompatible, fast electron transfer	ChOx, Urs, DNA, IgG
ZnO	9.5	Biocompatible, fast electron transfer, non-toxic, diverse nanomorphologies	ChOx, GOx, Urs, DNA, MIOs, IgG

**Table 2:** Categories of externally powered or self-powered chemical sensors [23,25,26,27,28,29,30,31,32,33,34].

Category	Matrix	Sensitivity	Respnse Time	Self-Life
EPCS	Tetraethylorthosilcate	–	15 s	8 weeks
EPCS	Controlled pore glass	0.1 mM	2 min	12 months
EPCS	Octadecyl silica	–	7–12 min	8 weeks
EPCS	Polyvinyl membrane/oxygen electrode	–	–	20 days
EPCS	BSA/polycarbonate/oxygen electrode	–	90 s	8 weeks
EPCS	Poly(aniline-co-pyrrole)/ITO	93.35 nA μM^−1^	–	10 weeks
EPCS	Cystamine	625.5 nA mM^−1^	3 s	5 days
EPCS	ZnO nanostructures	61.7 µA µM^−1^ cm^−2^	–	32 days
SPCS	ZnO nanowalls	53 mV/decade	5 s	3 weeks
SPCS	ZnO nanorods	35.2 mV/decade	10 s	12 weeks
SPCS	Co_3_O_4_ nanowires	−94 mV/decade	10 s	–
